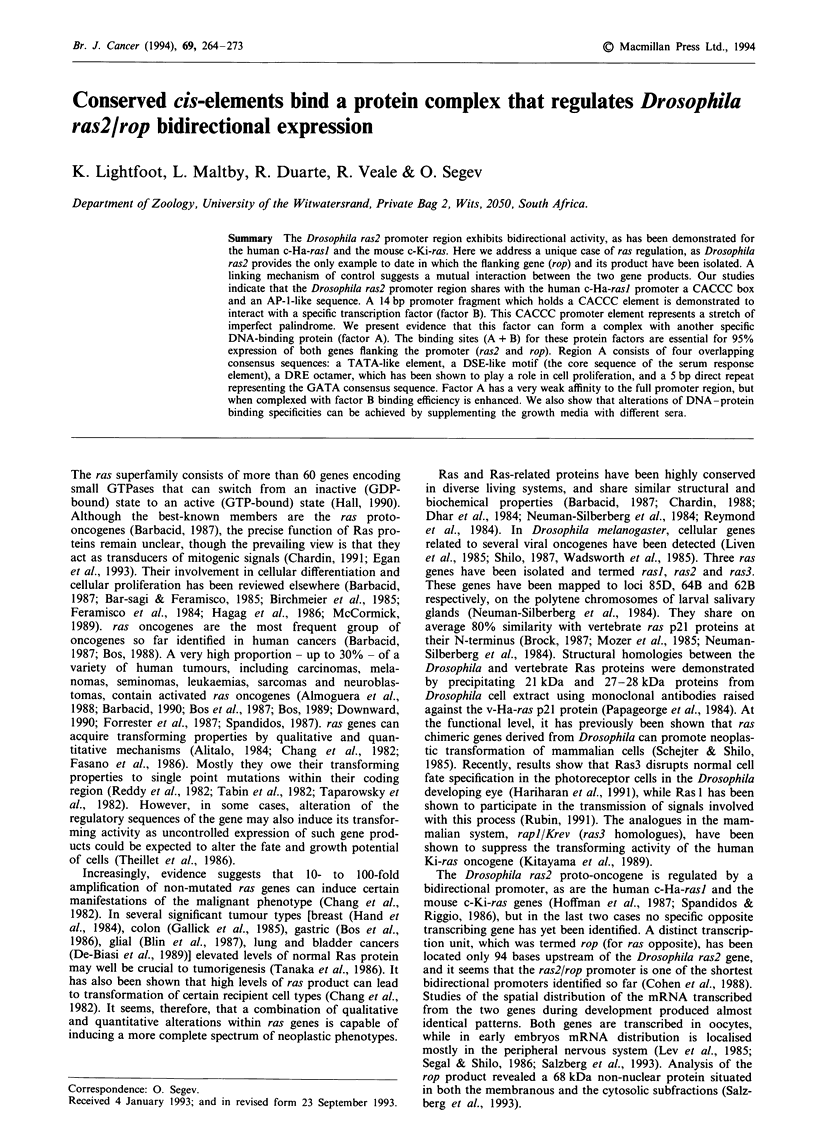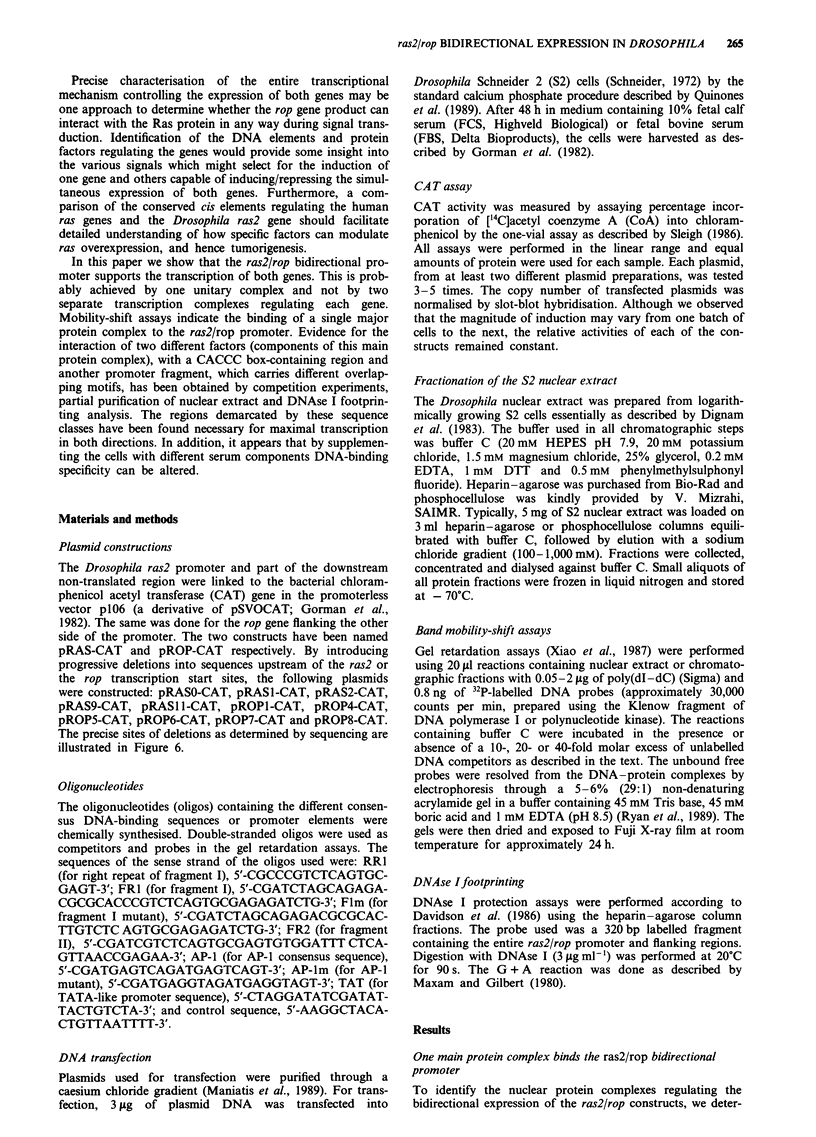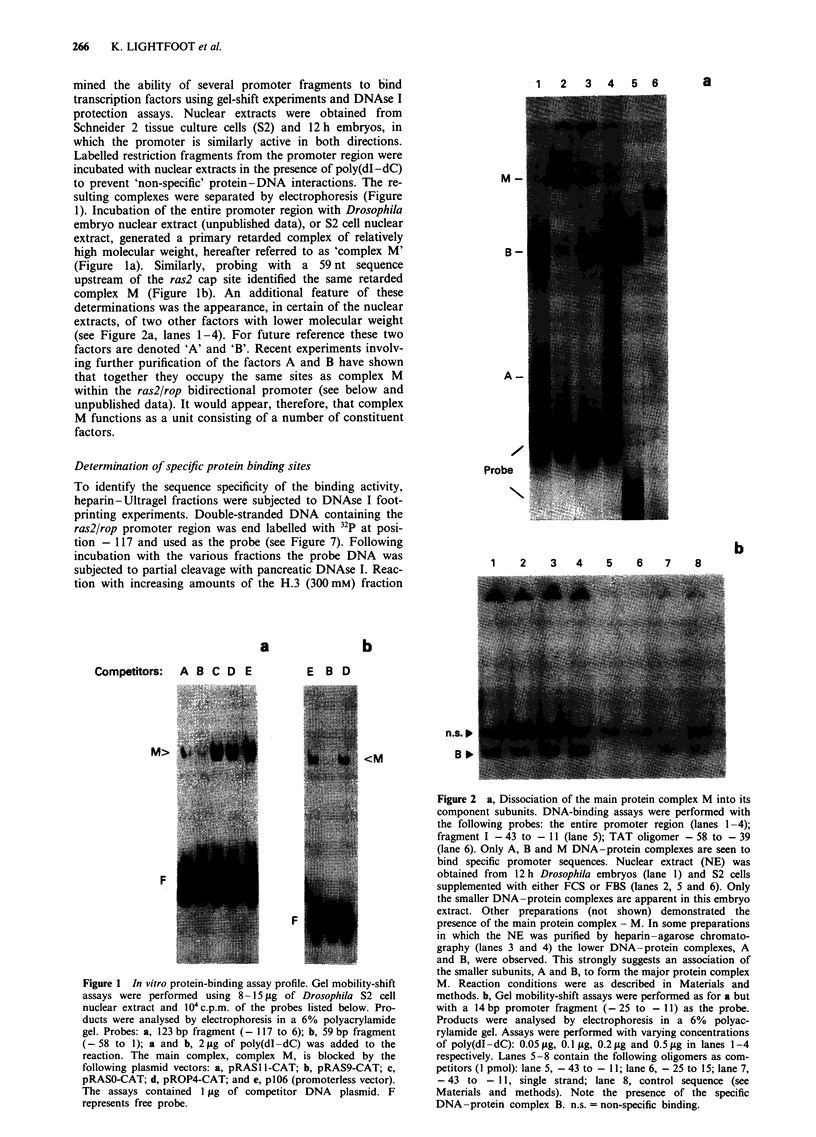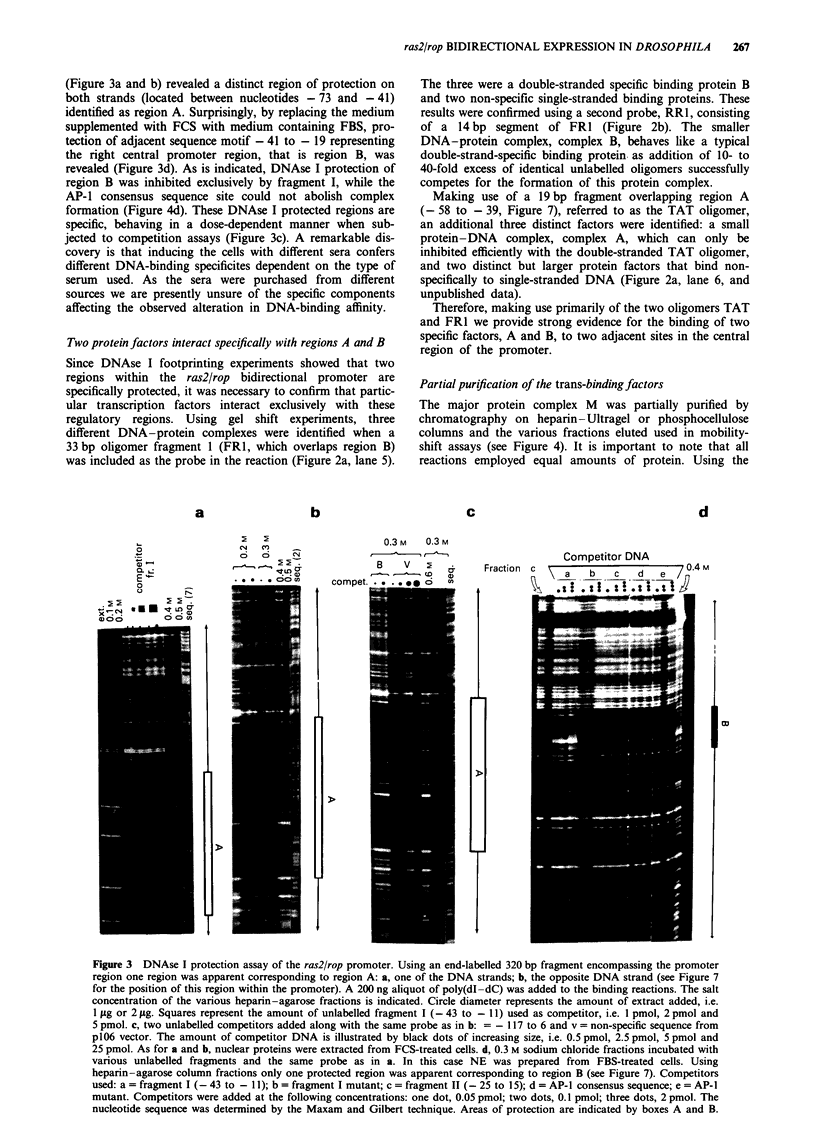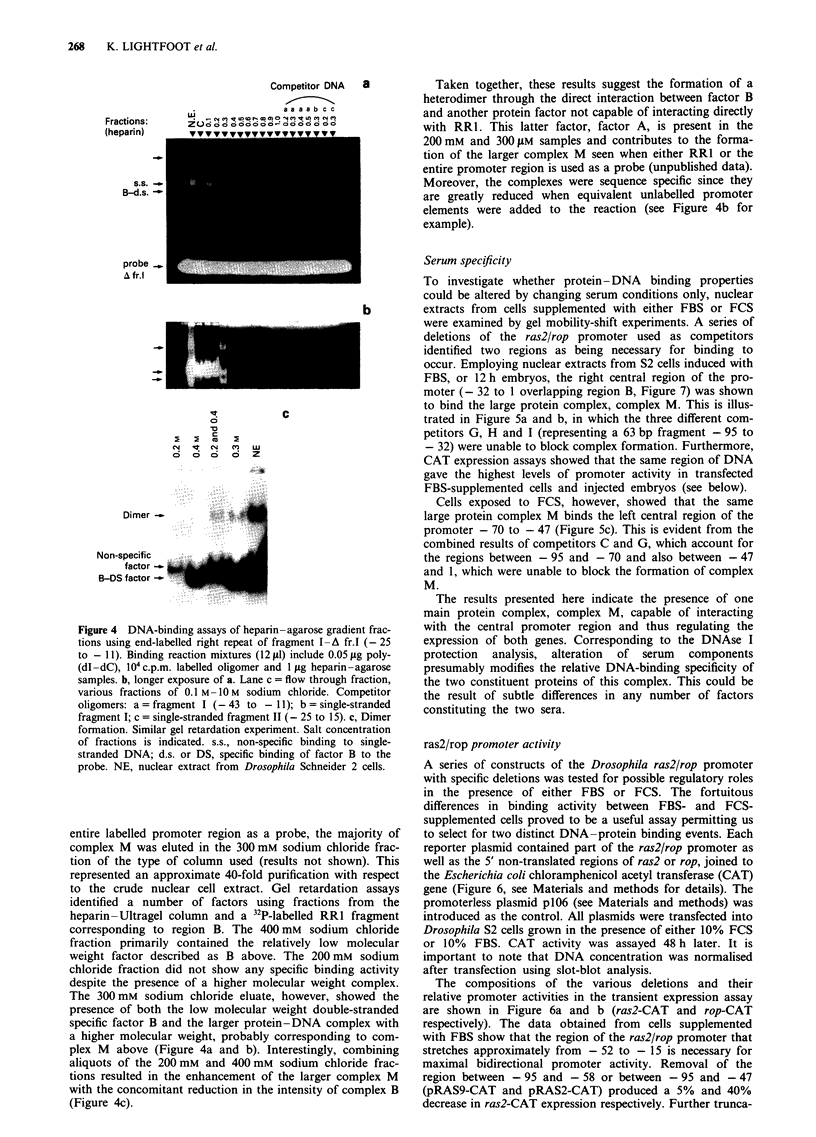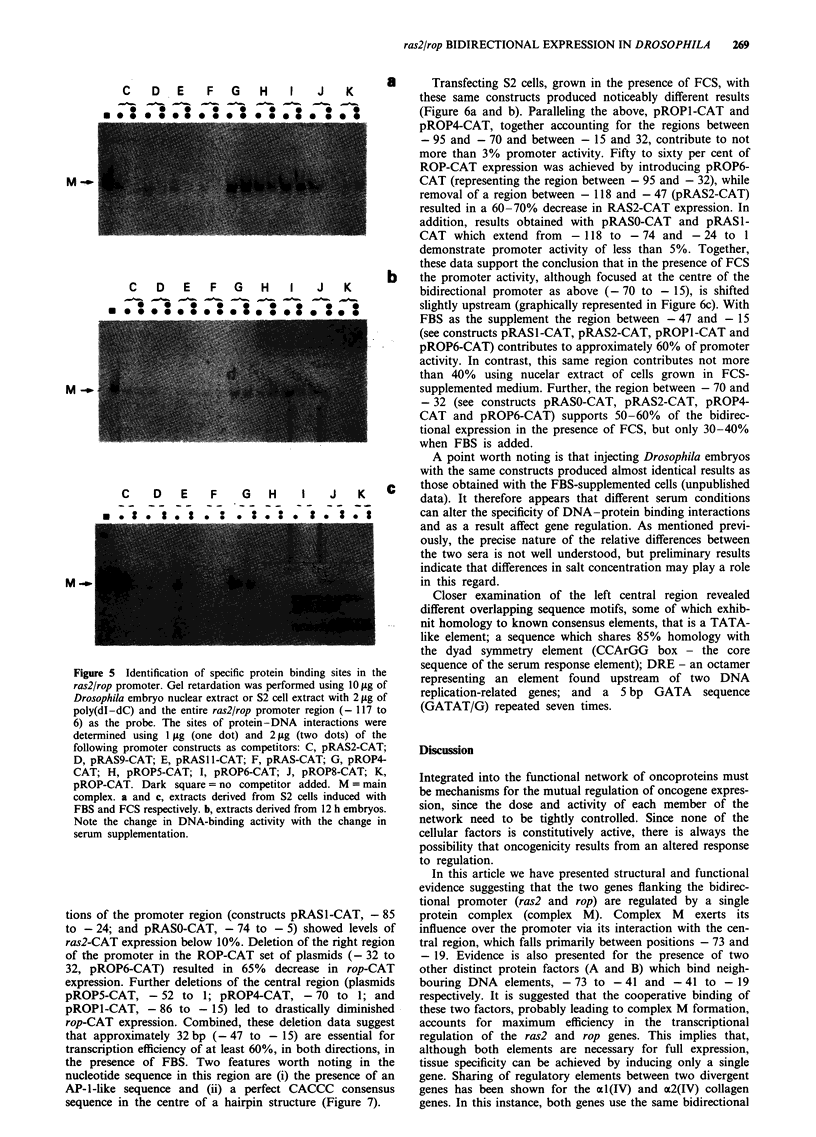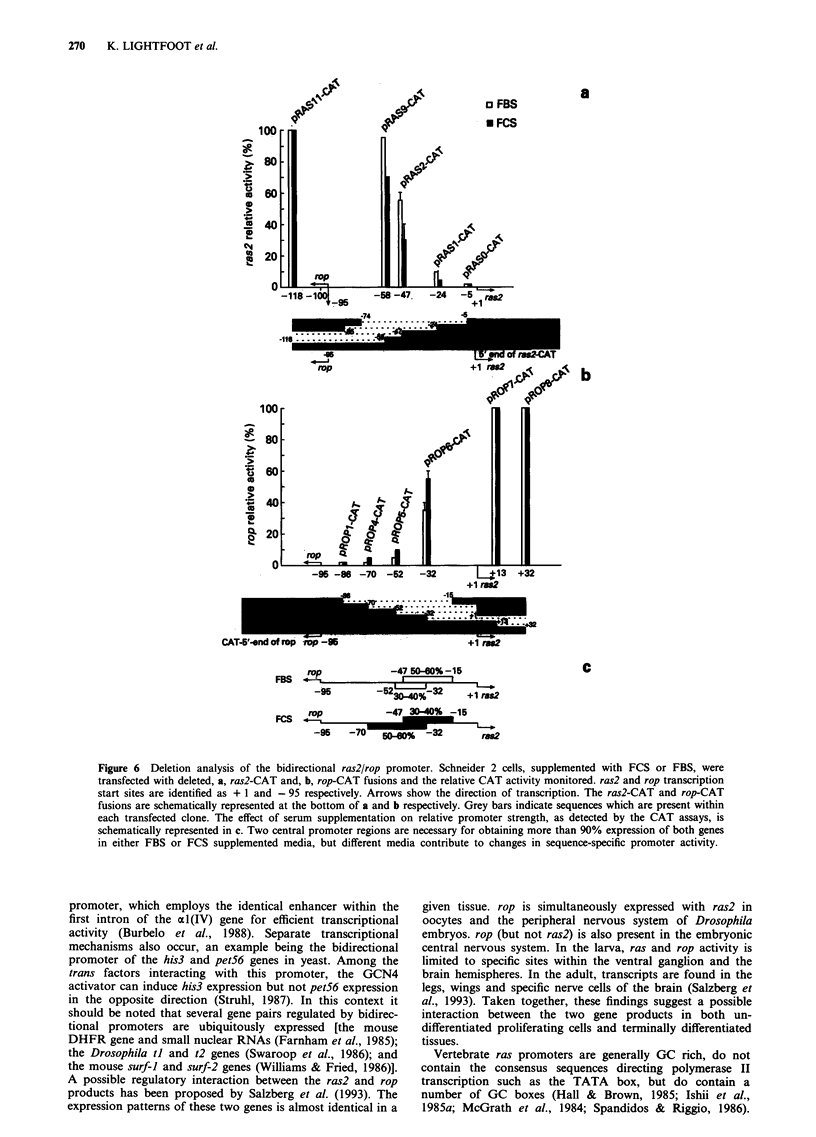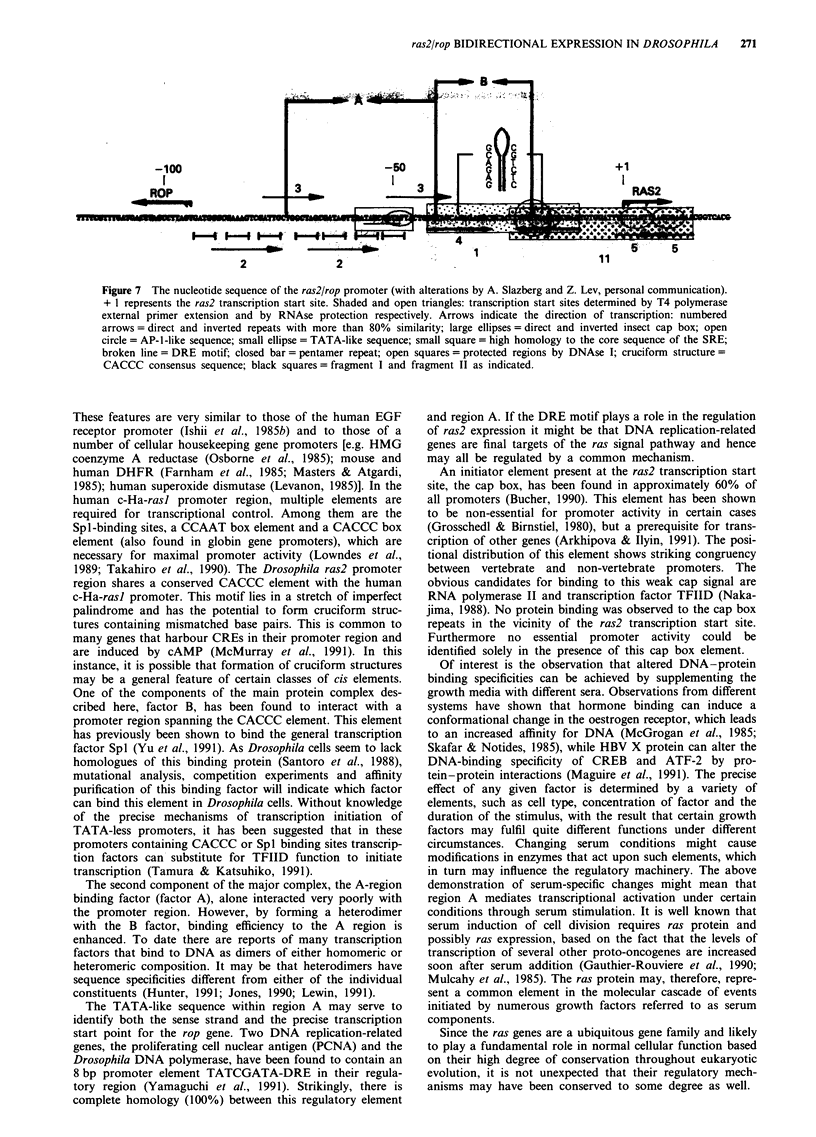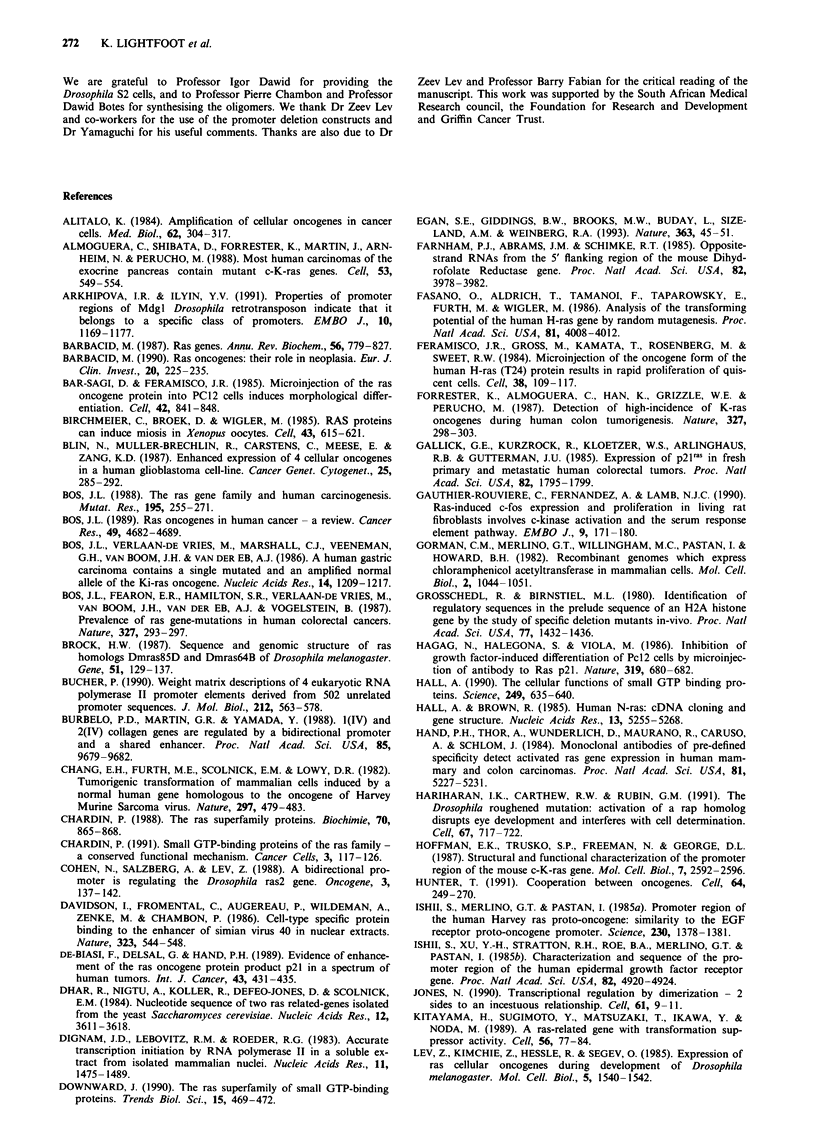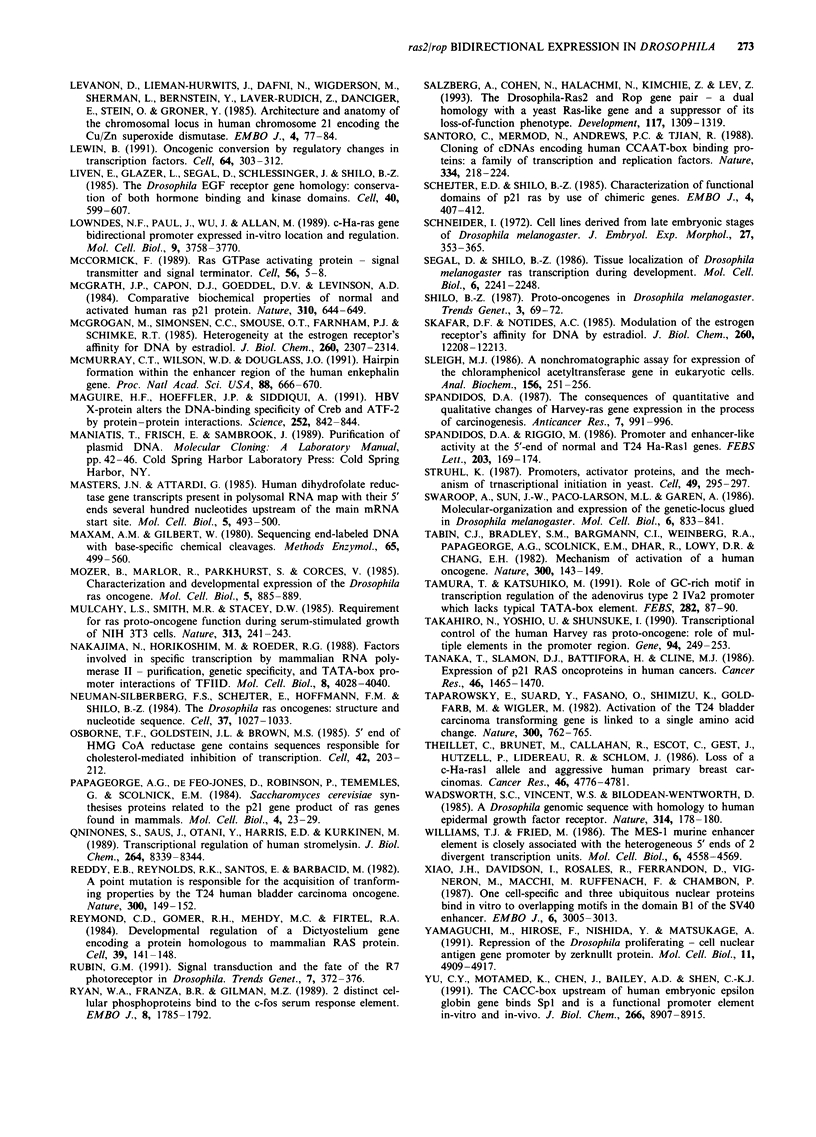# Conserved cis-elements bind a protein complex that regulates Drosophila ras2/rop bidirectional expression.

**DOI:** 10.1038/bjc.1994.50

**Published:** 1994-02

**Authors:** K. Lightfoot, L. Maltby, R. Duarte, R. Veale, O. Segev

**Affiliations:** Department of Zoology, University of the Witwatersrand, South Africa.

## Abstract

**Images:**


*Br. J. Cancer* (1994), **69**, 264-273 © Macmillan Press Ltd., 1994

### Conserved *cis*-elements bind a protein complex that regulates *Drosophila ras2/rop* bidirectional expression

K. Lightfoot, L. Maltby, R. Duarte, R. Veale & O. Segev

*Department of Zoology, University of the Witwatersrand, Private Bag 2, Wits, 2050, South Africa.*

**Summary** The *Drosophila ras2* promoter region exhibits bidirectional activity, as has been demonstrated for the human c-Ha-*ras1* and the mouse c-Ki-*ras*. Here we address a unique case of *ras* regulation, as *Drosophila ras2* provides the only example to date in which the flanking gene (*rop*) and its product have been isolated. A linking mechanism of control suggests a mutual interaction between the two gene products. Our studies indicate that the *Drosophila ras2* promoter region shares with the human c-Ha-*ras1* promoter a CACCC box and an AP-1-like sequence. A 14 bp promoter fragment which holds a CACCC element is demonstrated to interact with a specific transcription factor (factor B). This CACCC promoter element represents a stretch of imperfect palindrome. We present evidence that this factor can form a complex with another specific DNA-binding protein (factor A). The binding sites (A + B) for these protein factors are essential for 95% expression of both genes flanking the promoter (*ras2* and *rop*). Region A consists of four overlapping consensus sequences: a TATA-like element, a DSE-like motif (the core sequence of the serum response element), a DRE octamer, which has been shown to play a role in cell proliferation, and a 5 bp direct repeat representing the GATA consensus sequence. Factor A has a very weak affinity to the full promoter region, but when complexed with factor B binding efficiency is enhanced. We also show that alterations of DNA-protein binding specificities can be achieved by supplementing the growth media with different sera.

The *ras* superfamily consists of more than 60 genes encoding small GTPases that can switch from an inactive (GDP-bound) state to an active (GTP-bound) state (Hall, 1990). Although the best-known members are the *ras* proto-oncogenes (Barbacid, 1987), the precise function of Ras proteins remain unclear, though the prevailing view is that they act as transducers of mitogenic signals (Chardin, 1991; Egan *et al.*, 1993). Their involvement in cellular differentiation and cellular proliferation has been reviewed elsewhere (Barbacid, 1987; Bar-sagi & Feramisco, 1985; Birchmeier *et al.*, 1985; Feramisco *et al.*, 1984; Hagag *et al.*, 1986; McCormick, 1989). *ras* oncogenes are the most frequent group of oncogenes so far identified in human cancers (Barbacid, 1987; Bos, 1988). A very high proportion - up to 30% - of a variety of human tumours, including carcinomas, melanomas, seminomas, leukaemias, sarcomas and neuroblastomas, contain activated *ras* oncogenes (Almoguera *et al.*, 1988; Barbacid, 1990; Bos *et al.*, 1987; Bos, 1989; Downward, 1990; Forrester *et al.*, 1987; Spandidos, 1987). *ras* genes can acquire transforming properties by qualitative and quantitative mechanisms (Alitalo, 1984; Chang *et al.*, 1982; Fasano *et al.*, 1986). Mostly they owe their transforming properties to single point mutations within their coding region (Reddy *et al.*, 1982; Tabin *et al.*, 1982; Taparowsky *et al.*, 1982). However, in some cases, alteration of the regulatory sequences of the gene may also induce its transforming activity as uncontrolled expression of such gene products could be expected to alter the fate and growth potential of cells (Theillet *et al.*, 1986).

Increasingly, evidence suggests that 10- to 100-fold amplification of non-mutated *ras* genes can induce certain manifestations of the malignant phenotype (Chang *et al.*, 1982). In several significant tumour types [breast (Hand *et al.*, 1984), colon (Gallick *et al.*, 1985), gastric (Bos *et al.*, 1986), glial (Blin *et al.*, 1987), lung and bladder cancers (De-Biasi *et al.*, 1989)] elevated levels of normal Ras protein may well be crucial to tumorigenesis (Tanaka *et al.*, 1986). It has also been shown that high levels of *ras* product can lead to transformation of certain recipient cell types (Chang *et al.*, 1982). It seems, therefore, that a combination of qualitative and quantitative alterations within *ras* genes is capable of inducing a more complete spectrum of neoplastic phenotypes.

Ras and Ras-related proteins have been highly conserved in diverse living systems, and share similar structural and biochemical properties (Barbacid, 1987; Chardin, 1988; Dhar *et al.*, 1984; Neuman-Silberberg *et al.*, 1984; Reymond *et al.*, 1984). In *Drosophila melanogaster*, cellular genes related to several viral oncogenes have been detected (Liven *et al.*, 1985; Shilo, 1987, Wadsworth *et al.*, 1985). Three *ras* genes have been isolated and termed *ras1*, *ras2* and *ras3*. These genes have been mapped to loci 85D, 64B and 62B respectively, on the polytene chromosomes of larval salivary glands (Neuman-Silberberg *et al.*, 1984). They share on average 80% similarity with vertebrate *ras* p21 proteins at their N-terminus (Brock, 1987; Mozer *et al.*, 1985; Neuman-Silberberg *et al.*, 1984). Structural homologies between the *Drosophila* and vertebrate Ras proteins were demonstrated by precipitating 21 kDa and 27-28 kDa proteins from *Drosophila* cell extract using monoclonal antibodies raised against the v-Ha-*ras* p21 protein (Papageorge *et al.*, 1984). At the functional level, it has previously been shown that *ras* chimeric genes derived from *Drosophila* can promote neoplastic transformation of mammalian cells (Schejter & Shilo, 1985). Recently, results show that Ras3 disrupts normal cell fate specification in the photoreceptor cells in the *Drosophila* developing eye (Hariharan *et al.*, 1991), while Ras1 has been shown to participate in the transmission of signals involved with this process (Rubin, 1991). The analogues in the mammalian system, *rap1/Krev* (*ras3* homologues), have been shown to suppress the transforming activity of the human Ki-*ras* oncogene (Kitayama *et al.*, 1989).

The *Drosophila ras2* proto-oncogene is regulated by a bidirectional promoter, as are the human c-Ha-*ras1* and the mouse c-Ki-*ras* genes (Hoffman *et al.*, 1987; Spandidos & Riggio, 1986), but in the last two cases no specific opposite transcribing gene has yet been identified. A distinct transcription unit, which was termed *rop* (for *ras* opposite), has been located only 94 bases upstream of the *Drosophila ras2* gene, and it seems that the *ras2/rop* promoter is one of the shortest bidirectional promoters identified so far (Cohen *et al.*, 1988). Studies of the spatial distribution of the mRNA transcribed from the two genes during development produced almost identical patterns. Both genes are transcribed in oocytes, while in early embryos mRNA distribution is localised mostly in the peripheral nervous system (Lev *et al.*, 1985; Segal & Shilo, 1986; Salzberg *et al.*, 1993). Analysis of the *rop* product revealed a 68 kDa non-nuclear protein situated in both the membranous and the cytosolic subfractions (Salzberg *et al.*, 1993).

Correspondence: O. Segev.
Received 4 January 1993; and in revised form 23 September 1993.

Precise characterisation of the entire transcriptional mechanism controlling the expression of both genes may be one approach to determine whether the *rop* gene product can interact with the Ras protein in any way during signal transduction. Identification of the DNA elements and protein factors regulating the genes would provide some insight into the various signals which might select for the induction of one gene and others capable of inducing/repressing the simultaneous expression of both genes. Furthermore, a comparison of the conserved *cis* elements regulating the human *ras* genes and the *Drosophila ras2* gene should facilitate detailed understanding of how specific factors can modulate *ras* overexpression, and hence tumorigenesis.

In this paper we show that the *ras2/rop* bidirectional promoter supports the transcription of both genes. This is probably achieved by one unitary complex and not by two separate transcription complexes regulating each gene. Mobility-shift assays indicate the binding of a single major protein complex to the *ras2*/rop promoter. Evidence for the interaction of two different factors (components of this main protein complex), with a CACCC box-containing region and another promoter fragment, which carries different overlapping motifs, has been obtained by competition experiments, partial purification of nuclear extract and DNAse I footprinting analysis. The regions demarcated by these sequence classes have been found necessary for maximal transcription in both directions. In addition, it appears that by supplementing the cells with different serum components DNA-binding specificity can be altered.

### Materials and methods

### *Plasmid constructions*

The *Drosophila ras2* promoter and part of the downstream non-translated region were linked to the bacterial chloramphenicol acetyl transferase (CAT) gene in the promoterless vector p106 (a derivative of pSVOCAT; Gorman *et al.*, 1982). The same was done for the *rop* gene flanking the other side of the promoter. The two constructs have been named pRAS-CAT and pROP-CAT respectively. By introducing progressive deletions into sequences upstream of the *ras2* or the *rop* transcription start sites, the following plasmids were constructed: pRAS0-CAT, pRAS1-CAT, pRAS2-CAT, pRAS9-CAT, pRAS11-CAT, pROP1-CAT, pROP4-CAT, pROP5-CAT, pROP6-CAT, pROP7-CAT and pROP8-CAT. The precise sites of deletions as determined by sequencing are illustrated in Figure 6.

### *Oligonucleotides*

The oligonucleotides (oligos) containing the different consensus DNA-binding sequences or promoter elements were chemically synthesised. Double-stranded oligos were used as competitors and probes in the gel retardation assays. The sequences of the sense strand of the oligos used were: RR1 (for right repeat of fragment I), 5′-CGCCCGTCTCAGTGC-GAGT-3′; FR1 (for fragment I), 5′-CGATCTAGCAGAGA-CGCGCACCCGTCTCAGTGCGAGAGATCTG-3′; F1m (for fragment I mutant), 5′-CGATCTAGCAGAGACGCGCAC-TTGTCTC AGTGCGAGAGATCTG-3′; FR2 (for fragment II), 5′-CGATCGTCTCAGTGCGAGTGTGGATTT CTCA-GTTAACCGAGAA-3′; AP-1 (for AP-1 consensus sequence), 5′-CGATGAGTCAGATGAGTCAGT-3′; AP-1m (for AP-1 mutant), 5′-CGATGAGGTAGATGAGGTAGT-3′; TAT (for TATA-like promoter sequence), 5′-CTAGGATATCGATAT-TACTGTCTA-3′; and control sequence, 5′-AAGGCTACA-CTGTTAATTTT-3′.

### *DNA transfection*

Plasmids used for transfection were purified through a caesium chloride gradient (Maniatis *et al.*, 1989). For transfection, 3 μg of plasmid DNA was transfected into *Drosophila* Schneider 2 (S2) cells (Schneider, 1972) by the standard calcium phosphate procedure described by Quinones *et al.* (1989). After 48 h in medium containing 10% fetal calf serum (FCS, Highveld Biological) or fetal bovine serum (FBS, Delta Bioproducts), the cells were harvested as described by Gorman *et al.* (1982).

### *CAT assay*

CAT activity was measured by assaying percentage incorporation of [$^{14}$C]acetyl coenzyme A (CoA) into chloramphenicol by the one-vial assay as described by Sleigh (1986). All assays were performed in the linear range and equal amounts of protein were used for each sample. Each plasmid, from at least two different plasmid preparations, was tested 3–5 times. The copy number of transfected plasmids was normalised by slot-blot hybridisation. Although we observed that the magnitude of induction may vary from one batch of cells to the next, the relative activities of each of the constructs remained constant.

### *Fractionation of the S2 nuclear extract*

The *Drosophila* nuclear extract was prepared from logarithmically growing S2 cells essentially as described by Dignam *et al.* (1983). The buffer used in all chromatographic steps was buffer C (20 mM HEPES pH 7.9, 20 mM potassium chloride, 1.5 mM magnesium chloride, 25% glycerol, 0.2 mM EDTA, 1 mM DTT and 0.5 mM phenylmethylsulphonyl fluoride). Heparin–agarose was purchased from Bio-Rad and phosphocellulose was kindly provided by V. Mizrahi, SAIMR. Typically, 5 mg of S2 nuclear extract was loaded on 3 ml heparin–agarose or phosphocellulose columns equilibrated with buffer C, followed by elution with a sodium chloride gradient (100–1,000 mM). Fractions were collected, concentrated and dialysed against buffer C. Small aliquots of all protein fractions were frozen in liquid nitrogen and stored at − 70°C.

### *Band mobility-shift assays*

Gel retardation assays (Xiao *et al.*, 1987) were performed using 20 μl reactions containing nuclear extract or chromatographic fractions with 0.05–2 μg of poly(dI–dC) (Sigma) and 0.8 ng of $^{32}$P-labelled DNA probes (approximately 30,000 counts per min, prepared using the Klenow fragment of DNA polymerase I or polynucleotide kinase). The reactions containing buffer C were incubated in the presence or absence of a 10-, 20- or 40-fold molar excess of unlabelled DNA competitors as described in the text. The unbound free probes were resolved from the DNA–protein complexes by electrophoresis through a 5–6% (29:1) non-denaturing acrylamide gel in a buffer containing 45 mM Tris base, 45 mM boric acid and 1 mM EDTA (pH 8.5) (Ryan *et al.*, 1989). The gels were then dried and exposed to Fuji X-ray film at room temperature for approximately 24 h.

### *DNAse I footprinting*

DNAse I protection assays were performed according to Davidson *et al.* (1986) using the heparin–agarose column fractions. The probe used was a 320 bp labelled fragment containing the entire *ras2/rop* promoter and flanking regions. Digestion with DNAse I (3 μg ml$^{-1}$) was performed at 20°C for 90 s. The G + A reaction was done as described by Maxam and Gilbert (1980).

### Results

### *One main protein complex binds the* ras2/rop *bidirectional promoter*

To identify the nuclear protein complexes regulating the bidirectional expression of the *ras2/rop* constructs, we determined the ability of several promoter fragments to bind transcription factors using gel-shift experiments and DNAse I protection assays. Nuclear extracts were obtained from Schneider 2 tissue culture cells (S2) and 12 h embryos, in which the promoter is similarly active in both directions. Labelled restriction fragments from the promoter region were incubated with nuclear extracts in the presence of poly(dI–dC) to prevent 'non-specific' protein–DNA interactions. The resulting complexes were separated by electrophoresis (Figure 1). Incubation of the entire promoter region with *Drosophila* embryo nuclear extract (unpublished data), or S2 cell nuclear extract, generated a primary retarded complex of relatively high molecular weight, hereafter referred to as 'complex M' (Figure 1a). Similarly, probing with a 59 nt sequence upstream of the *ras2* cap site identified the same retarded complex M (Figure 1b). An additional feature of these determinations was the appearance, in certain of the nuclear extracts, of two other factors with lower molecular weight (see Figure 2a, lanes 1–4). For future reference these two factors are denoted 'A' and 'B'. Recent experiments involving further purification of the factors A and B have shown that together they occupy the same sites as complex M within the *ras2/rop* bidirectional promoter (see below and unpublished data). It would appear, therefore, that complex M functions as a unit consisting of a number of constituent factors.

### *Determination of specific protein binding sites*

To identify the sequence specificity of the binding activity, heparin–Ultragel fractions were subjected to DNAse I footprinting experiments. Double-stranded DNA containing the *ras2/rop* promoter region was end labelled with $^{32}P$ at position − 117 and used as the probe (see Figure 7). Following incubation with the various fractions the probe DNA was subjected to partial cleavage with pancreatic DNAse I. Reaction with increasing amounts of the H.3 (300 mM) fraction

**Figure 1** *In vitro* protein-binding assay profile. Gel mobility-shift assays were performed using 8–15 µg of *Drosophila* S2 cell nuclear extract and $10^4$ c.p.m. of the probes listed below. Products were analysed by electrophoresis in a 6% polyacrylamide gel. Probes: **a**, 123 bp fragment (− 117 to 6); **b**, 59 bp fragment (− 58 to 1); **a** and **b**, 2 µg of poly(dI–dC) was added to the reaction. The main complex, complex M, is blocked by the following plasmid vectors: **a**, pRAS11-CAT; **b**, pRAS9-CAT; **c**, pRAS0-CAT; **d**, pROP4-CAT; and **e**, p106 (promoterless vector). The assays contained 1 µg of competitor DNA plasmid. F represents free probe.

**Figure 2** **a**, Dissociation of the main protein complex M into its component subunits. DNA-binding assays were performed with the following probes: the entire promoter region (lanes 1–4); fragment I − 43 to − 11 (lane 5); TAT oligomer − 58 to − 39 (lane 6). Only A, B and M DNA–protein complexes are seen to bind specific promoter sequences. Nuclear extract (NE) was obtained from 12 h *Drosophila* embryos (lane 1) and S2 cells supplemented with either FCS or FBS (lanes 2, 5 and 6). Only the smaller DNA–protein complexes are apparent in this embryo extract. Other preparations (not shown) demonstrated the presence of the main protein complex – M. In some preparations in which the NE was purified by heparin-agarose chromatography (lanes 3 and 4) the lower DNA–protein complexes, A and B, were observed. This strongly suggests an association of the smaller subunits, A and B, to form the major protein complex M. Reaction conditions were as described in Materials and methods. **b**, Gel mobility-shift assays were performed as for **a** but with a 14 bp promoter fragment (− 25 to − 11) as the probe. Products were analysed by electrophoresis in a 6% polyacrylamide gel. Assays were performed with varying concentrations of poly(dI–dC): 0.05 µg, 0.1 µg, 0.2 µg and 0.5 µg in lanes 1–4 respectively. Lanes 5–8 contain the following oligomers as competitors (1 pmol): lane 5, − 43 to − 11; lane 6, − 25 to 15; lane 7, − 43 to − 11, single strand; lane 8, control sequence (see Materials and methods). Note the presence of the specific DNA–protein complex B. n.s. = non-specific binding.

(Figure 3a and b) revealed a distinct region of protection on both strands (located between nucleotides − 73 and − 41) identified as region A. Surprisingly, by replacing the medium supplemented with FCS with medium containing FBS, protection of adjacent sequence motif − 41 to − 19 representing the right central promoter region, that is region B, was revealed (Figure 3d). As is indicated, DNAse I protection of region B was inhibited exclusively by fragment I, while the AP-1 consensus sequence site could not abolish complex formation (Figure 4d). These DNAse I protected regions are specific, behaving in a dose-dependent manner when subjected to competition assays (Figure 3c). A remarkable discovery is that inducing the cells with different sera confers different DNA-binding specificites dependent on the type of serum used. As the sera were purchased from different sources we are presently unsure of the specific components affecting the observed alteration in DNA-binding affinity.

### *Two protein factors interact specifically with regions A and B*

Since DNAse I footprinting experiments showed that two regions within the *ras2/rop* bidirectional promoter are specifically protected, it was necessary to confirm that particular transcription factors interact exclusively with these regulatory regions. Using gel shift experiments, three different DNA–protein complexes were identified when a 33 bp oligomer fragment 1 (FR1, which overlaps region B) was included as the probe in the reaction (Figure 2a, lane 5). The three were a double-stranded specific binding protein B and two non-specific single-stranded binding proteins. These results were confirmed using a second probe, RR1, consisting of a 14 bp segment of FR1 (Figure 2b). The smaller DNA–protein complex, complex B, behaves like a typical double-strand-specific binding protein as addition of 10- to 40-fold excess of identical unlabelled oligomers successfully competes for the formation of this protein complex.

Making use of a 19 bp fragment overlapping region A (− 58 to − 39, Figure 7), referred to as the TAT oligomer, an additional three distinct factors were identified: a small protein–DNA complex, complex A, which can only be inhibited efficiently with the double-stranded TAT oligomer, and two distinct but larger protein factors that bind non-specifically to single-stranded DNA (Figure 2a, lane 6, and unpublished data).

Therefore, making use primarily of the two oligomers TAT and FR1 we provide strong evidence for the binding of two specific factors, A and B, to two adjacent sites in the central region of the promoter.

### *Partial purification of the* trans-*binding factors*

The major protein complex M was partially purified by chromatography on heparin–Ultragel or phosphocellulose columns and the various fractions eluted used in mobility-shift assays (see Figure 4). It is important to note that all reactions employed equal amounts of protein. Using the

**Figure 3** DNAse I protection assay of the *ras2/rop* promoter. Using an end-labelled 320 bp fragment encompassing the promoter region one region was apparent corresponding to region A: **a**, one of the DNA strands; **b**, the opposite DNA strand (see Figure 7 for the position of this region within the promoter). A 200 ng aliquot of poly(dI–dC) was added to the binding reactions. The salt concentration of the various heparin–agarose fractions is indicated. Circle diameter represents the amount of extract added, i.e. 1 μg or 2 μg. Squares represent the amount of unlabelled fragment I (− 43 to − 11) used as competitor, i.e. 1 pmol, 2 pmol and 5 pmol. **c**, two unlabelled competitors added along with the same probe as in **b**: = − 117 to 6 and v = non-specific sequence from p106 vector. The amount of competitor DNA is illustrated by black dots of increasing size, i.e. 0.5 pmol, 2.5 pmol, 5 pmol and 25 pmol. As for **a** and **b**, nuclear proteins were extracted from FCS-treated cells. **d**, 0.3 M sodium chloride fractions incubated with various unlabelled fragments and the same probe as in **a**. In this case NE was prepared from FBS-treated cells. Using heparin–agarose column fractions only one protected region was apparent corresponding to region B (see Figure 7). Competitors used: a = fragment I (− 43 to − 11); b = fragment I mutant; c = fragment II (− 25 to 15); d = AP-1 consensus sequence; e = AP-1 mutant. Competitors were added at the following concentrations: one dot, 0.05 pmol; two dots, 0.1 pmol; three dots, 2 pmol. The nucleotide sequence was determined by the Maxam and Gilbert technique. Areas of protection are indicated by boxes A and B.

**Figure 4** DNA-binding assays of heparin–agarose gradient fractions using end-labelled right repeat of fragment I–Δ fr.I (– 25 to – 11). Binding reaction mixtures (12 μl) include 0.05 μg poly-(dI–dC), $10^4$ c.p.m. labelled oligomer and 1 μg heparin–agarose samples. **b**, longer exposure of **a**. Lane c = flow through fraction, various fractions of 0.1 M–10 M sodium chloride. Competitor oligomers: a = fragment I (– 43 to – 11); b = single-stranded fragment I; c = single-stranded fragment II (– 25 to 15). **c**, Dimer formation. Similar gel retardation experiment. Salt concentration of fractions is indicated. s.s., non-specific binding to single-stranded DNA; d.s. or DS, specific binding of factor B to the probe. NE, nuclear extract from *Drosophila* Schneider 2 cells.

entire labelled promoter region as a probe, the majority of complex M was eluted in the 300 mM sodium chloride fraction of the type of column used (results not shown). This represented an approximate 40-fold purification with respect to the crude nuclear cell extract. Gel retardation assays identified a number of factors using fractions from the heparin–Ultragel column and a $^{32}$P-labelled RR1 fragment corresponding to region B. The 400 mM sodium chloride fraction primarily contained the relatively low molecular weight factor described as B above. The 200 mM sodium chloride fraction did not show any specific binding activity despite the presence of a higher molecular weight complex. The 300 mM sodium chloride eluate, however, showed the presence of both the low molecular weight double-stranded specific factor B and the larger protein–DNA complex with a higher molecular weight, probably corresponding to complex M above (Figure 4a and b). Interestingly, combining aliquots of the 200 mM and 400 mM sodium chloride fractions resulted in the enhancement of the larger complex M with the concomitant reduction in the intensity of complex B (Figure 4c).

Taken together, these results suggest the formation of a heterodimer through the direct interaction between factor B and another protein factor not capable of interacting directly with RR1. This latter factor, factor A, is present in the 200 mM and 300 μM samples and contributes to the formation of the larger complex M seen when either RR1 or the entire promoter region is used as a probe (unpublished data). Moreover, the complexes were sequence specific since they are greatly reduced when equivalent unlabelled promoter elements were added to the reaction (see Figure 4b for example).

### *Serum specificity*

To investigate whether protein–DNA binding properties could be altered by changing serum conditions only, nuclear extracts from cells supplemented with either FBS or FCS were examined by gel mobility-shift experiments. A series of deletions of the *ras2/rop* promoter used as competitors identified two regions as being necessary for binding to occur. Employing nuclear extracts from S2 cells induced with FBS, or 12 h embryos, the right central region of the promoter (– 32 to 1 overlapping region B, Figure 7) was shown to bind the large protein complex, complex M. This is illustrated in Figure 5a and b, in which the three different competitors G, H and I (representing a 63 bp fragment – 95 to – 32) were unable to block complex formation. Furthermore, CAT expression assays showed that the same region of DNA gave the highest levels of promoter activity in transfected FBS-supplemented cells and injected embryos (see below).

Cells exposed to FCS, however, showed that the same large protein complex M binds the left central region of the promoter – 70 to – 47 (Figure 5c). This is evident from the combined results of competitors C and G, which account for the regions between – 95 and – 70 and also between – 47 and 1, which were unable to block the formation of complex M.

The results presented here indicate the presence of one main protein complex, complex M, capable of interacting with the central promoter region and thus regulating the expression of both genes. Corresponding to the DNAse I protection analysis, alteration of serum components presumably modifies the relative DNA-binding specificity of the two constituent proteins of this complex. This could be the result of subtle differences in any number of factors constituting the two sera.

### ras2/rop *promoter activity*

A series of constructs of the *Drosophila ras2/rop* promoter with specific deletions was tested for possible regulatory roles in the presence of either FBS or FCS. The fortuitous differences in binding activity between FBS- and FCS-supplemented cells proved to be a useful assay permitting us to select for two distinct DNA–protein binding events. Each reporter plasmid contained part of the *ras2/rop* promoter as well as the 5′ non-translated regions of *ras2* or *rop*, joined to the *Escherichia coli* chloramphenicol acetyl transferase (CAT) gene (Figure 6, see Materials and methods for details). The promoterless plasmid p106 (see Materials and methods) was introduced as the control. All plasmids were transfected into *Drosophila* S2 cells grown in the presence of either 10% FCS or 10% FBS. CAT activity was assayed 48 h later. It is important to note that DNA concentration was normalised after transfection using slot-blot analysis.

The compositions of the various deletions and their relative promoter activities in the transient expression assay are shown in Figure 6a and b (*ras2*-CAT and *rop*-CAT respectively). The data obtained from cells supplemented with FBS show that the region of the *ras2/rop* promoter that stretches approximately from – 52 to – 15 is necessary for maximal bidirectional promoter activity. Removal of the region between – 95 and – 58 or between – 95 and – 47 (pRAS9-CAT and pRAS2-CAT) produced a 5% and 40% decrease in *ras2*-CAT expression respectively. Further trunca-

**Figure 5** Identification of specific protein binding sites in the *ras2/rop* promoter. Gel retardation was performed using 10 μg of *Drosophila* embryo nuclear extract or S2 cell extract with 2 μg of poly(dI–dC) and the entire *ras2/rop* promoter region (– 117 to 6) as the probe. The sites of protein–DNA interactions were determined using 1 μg (one dot) and 2 μg (two dots) of the following promoter constructs as competitors: C, pRAS2-CAT; D, pRAS9-CAT; E, pRAS11-CAT; F, pRAS-CAT; G, pROP4-CAT; H, pROP5-CAT; I, pROP6-CAT; J, pROP8-CAT; K, pROP-CAT. Dark square = no competitor added. M = main complex. **a** and **c**, extracts derived from S2 cells induced with FBS and FCS respectively. **b**, extracts derived from 12 h embryos. Note the change in DNA-binding activity with the change in serum supplementation.

tions of the promoter region (constructs pRAS1-CAT, – 85 to – 24; and pRAS0-CAT, – 74 to – 5) showed levels of *ras2*-CAT expression below 10%. Deletion of the right region of the promoter in the ROP-CAT set of plasmids (– 32 to 32, pROP6-CAT) resulted in 65% decrease in *rop*-CAT expression. Further deletions of the central region (plasmids pROP5-CAT, – 52 to 1; pROP4-CAT, – 70 to 1; and pROP1-CAT, – 86 to – 15) led to drastically diminished *rop*-CAT expression. Combined, these deletion data suggest that approximately 32 bp (– 47 to – 15) are essential for transcription efficiency of at least 60%, in both directions, in the presence of FBS. Two features worth noting in the nucleotide sequence in this region are (i) the presence of an AP-1-like sequence and (ii) a perfect CACCC consensus sequence in the centre of a hairpin structure (Figure 7).

Transfecting S2 cells, grown in the presence of FCS, with these same constructs produced noticeably different results (Figure 6a and b). Paralleling the above, pROP1-CAT and pROP4-CAT, together accounting for the regions between – 95 and – 70 and between – 15 and 32, contribute to not more than 3% promoter activity. Fifty to sixty per cent of ROP-CAT expression was achieved by introducing pROP6-CAT (representing the region between – 95 and – 32), while removal of a region between – 118 and – 47 (pRAS2-CAT) resulted in a 60–70% decrease in RAS2-CAT expression. In addition, results obtained with pRAS0-CAT and pRAS1-CAT which extend from – 118 to – 74 and – 24 to 1 demonstrate promoter activity of less than 5%. Together, these data support the conclusion that in the presence of FCS the promoter activity, although focused at the centre of the bidirectional promoter as above (– 70 to – 15), is shifted slightly upstream (graphically represented in Figure 6c). With FBS as the supplement the region between – 47 and – 15 (see constructs pRAS1-CAT, pRAS2-CAT, pROP1-CAT and pROP6-CAT) contributes to approximately 60% of promoter activity. In contrast, this same region contributes not more than 40% using nucelar extract of cells grown in FCS-supplemented medium. Further, the region between – 70 and – 32 (see constructs pRAS0-CAT, pRAS2-CAT, pROP4-CAT and pROP6-CAT) supports 50–60% of the bidirectional expression in the presence of FCS, but only 30–40% when FBS is added.

A point worth noting is that injecting *Drosophila* embryos with the same constructs produced almost identical results as those obtained with the FBS-supplemented cells (unpublished data). It therefore appears that different serum conditions can alter the specificity of DNA–protein binding interactions and as a result affect gene regulation. As mentioned previously, the precise nature of the relative differences between the two sera is not well understood, but preliminary results indicate that differences in salt concentration may play a role in this regard.

Closer examination of the left central region revealed different overlapping sequence motifs, some of which exhibnit homology to known consensus elements, that is a TATA-like element; a sequence which shares 85% homology with the dyad symmetry element (CCArGG box – the core sequence of the serum response element); DRE – an octamer representing an element found upstream of two DNA replication-related genes; and a 5 bp GATA sequence (GATAT/G) repeated seven times.

### Discussion

Integrated into the functional network of oncoproteins must be mechanisms for the mutual regulation of oncogene expression, since the dose and activity of each member of the network need to be tightly controlled. Since none of the cellular factors is constitutively active, there is always the possibility that oncogenicity results from an altered response to regulation.

In this article we have presented structural and functional evidence suggesting that the two genes flanking the bidirectional promoter (*ras2* and *rop*) are regulated by a single protein complex (complex M). Complex M exerts its influence over the promoter via its interaction with the central region, which falls primarily between positions – 73 and – 19. Evidence is also presented for the presence of two other distinct protein factors (A and B) which bind neighbouring DNA elements, – 73 to – 41 and – 41 to – 19 respectively. It is suggested that the cooperative binding of these two factors, probably leading to complex M formation, accounts for maximum efficiency in the transcriptional regulation of the *ras2* and *rop* genes. This implies that, although both elements are necessary for full expression, tissue specificity can be achieved by inducing only a single gene. Sharing of regulatory elements between two divergent genes has been shown for the α1(IV) and α2(IV) collagen genes. In this instance, both genes use the same bidirectional

**Figure 6** Deletion analysis of the bidirectional *ras2*/*rop* promoter. Schneider 2 cells, supplemented with FCS or FBS, were transfected with deleted, **a**, *ras2*-CAT and, **b**, *rop*-CAT fusions and the relative CAT activity monitored. *ras2* and *rop* transcription start sites are identified as + 1 and − 95 respectively. Arrows show the direction of transcription. The *ras2*-CAT and *rop*-CAT fusions are schematically represented at the bottom of **a** and **b** respectively. Grey bars indicate sequences which are present within each transfected clone. The effect of serum supplementation on relative promoter strength, as detected by the CAT assays, is schematically represented in **c**. Two central promoter regions are necessary for obtaining more than 90% expression of both genes in either FBS or FCS supplemented media, but different media contribute to changes in sequence-specific promoter activity.

promoter, which employs the identical enhancer within the first intron of the α1(IV) gene for efficient transcriptional activity (Burbelo *et al.*, 1988). Separate transcriptional mechanisms also occur, an example being the bidirectional promoter of the *his3* and *pet56* genes in yeast. Among the *trans* factors interacting with this promoter, the GCN4 activator can induce *his3* expression but not *pet56* expression in the opposite direction (Struhl, 1987). In this context it should be noted that several gene pairs regulated by bidirectional promoters are ubiquitously expressed [the mouse DHFR gene and small nuclear RNAs (Farnham *et al.*, 1985); the *Drosophila t1* and *t2* genes (Swaroop *et al.*, 1986); and the mouse *surf-1* and *surf-2* genes (Williams & Fried, 1986)]. A possible regulatory interaction between the *ras2* and *rop* products has been proposed by Salzberg *et al.* (1993). The expression patterns of these two genes is almost identical in a given tissue. *rop* is simultaneously expressed with *ras2* in oocytes and the peripheral nervous system of *Drosophila* embryos. *rop* (but not *ras2*) is also present in the embryonic central nervous system. In the larva, *ras* and *rop* activity is limited to specific sites within the ventral ganglion and the brain hemispheres. In the adult, transcripts are found in the legs, wings and specific nerve cells of the brain (Salzberg *et al.*, 1993). Taken together, these findings suggest a possible interaction between the two gene products in both undifferentiated proliferating cells and terminally differentiated tissues.

Vertebrate *ras* promoters are generally GC rich, do not contain the consensus sequences directing polymerase II transcription such as the TATA box, but do contain a number of GC boxes (Hall & Brown, 1985; Ishii *et al.*, 1985*a*; McGrath *et al.*, 1984; Spandidos & Riggio, 1986).

**Figure 7** The nucleotide sequence of the *ras2/rop* promoter (with alterations by A. Slazberg and Z. Lev, personal communication). + 1 represents the *ras2* transcription start site. Shaded and open triangles: transcription start sites determined by T4 polymerase external primer extension and by RNAse protection respectively. Arrows indicate the direction of transcription: numbered arrows = direct and inverted repeats with more than 80% similarity; large ellipses = direct and inverted insect cap box; open circle = AP-1-like sequence; small ellipse = TATA-like sequence; small square = high homology to the core sequence of the SRE; broken line = DRE motif; closed bar = pentamer repeat; open squares = protected regions by DNAse I; cruciform structure = CACCC consensus sequence; black squares = fragment I and fragment II as indicated.

These features are very similar to those of the human EGF receptor promoter (Ishii *et al.*, 1985*b*) and to those of a number of cellular housekeeping gene promoters [e.g. HMG coenzyme A reductase (Osborne *et al.*, 1985); mouse and human DHFR (Farnham *et al.*, 1985; Masters & Atgardi, 1985); human superoxide dismutase (Levanon, 1985)]. In the human c-Ha-*ras1* promoter region, multiple elements are required for transcriptional control. Among them are the Sp1-binding sites, a CCAAT box element and a CACCC box element (also found in globin gene promoters), which are necessary for maximal promoter activity (Lowndes *et al.*, 1989; Takahiro *et al.*, 1990). The *Drosophila ras2* promoter region shares a conserved CACCC element with the human c-Ha-*ras1* promoter. This motif lies in a stretch of imperfect palindrome and has the potential to form cruciform structures containing mismatched base pairs. This is common to many genes that harbour CREs in their promoter region and are induced by cAMP (McMurray *et al.*, 1991). In this instance, it is possible that formation of cruciform structures may be a general feature of certain classes of *cis* elements. One of the components of the main protein complex described here, factor B, has been found to interact with a promoter region spanning the CACCC element. This element has previously been shown to bind the general transcription factor Sp1 (Yu *et al.*, 1991). As *Drosophila* cells seem to lack homologues of this binding protein (Santoro *et al.*, 1988), mutational analysis, competition experiments and affinity purification of this binding factor will indicate which factor can bind this element in *Drosophila* cells. Without knowledge of the precise mechanisms of transcription initiation of TATA-less promoters, it has been suggested that in these promoters containing CACCC or Sp1 binding sites transcription factors can substitute for TFIID function to initiate transcription (Tamura & Katsuhiko, 1991).

The second component of the major complex, the A-region binding factor (factor A), alone interacted very poorly with the promoter region. However, by forming a heterodimer with the B factor, binding efficiency to the A region is enhanced. To date there are reports of many transcription factors that bind to DNA as dimers of either homomeric or heteromeric composition. It may be that heterodimers have sequence specificities different from either of the individual constituents (Hunter, 1991; Jones, 1990; Lewin, 1991).

The TATA-like sequence within region A may serve to identify both the sense strand and the precise transcription start point for the *rop* gene. Two DNA replication-related genes, the proliferating cell nuclear antigen (PCNA) and the *Drosophila* DNA polymerase, have been found to contain an 8 bp promoter element TATCGATA-DRE in their regulatory region (Yamaguchi *et al.*, 1991). Strikingly, there is complete homology (100%) between this regulatory element and region A. If the DRE motif plays a role in the regulation of *ras2* expression it might be that DNA replication-related genes are final targets of the *ras* signal pathway and hence may all be regulated by a common mechanism.

An initiator element present at the *ras2* transcription start site, the cap box, has been found in approximately 60% of all promoters (Bucher, 1990). This element has been shown to be non-essential for promoter activity in certain cases (Grosschedl & Birnstiel, 1980), but a prerequisite for transcription of other genes (Arkhipova & Ilyin, 1991). The positional distribution of this element shows striking congruency between vertebrate and non-vertebrate promoters. The obvious candidates for binding to this weak cap signal are RNA polymerase II and transcription factor TFIID (Nakajima, 1988). No protein binding was observed to the cap box repeats in the vicinity of the *ras2* transcription start site. Furthermore no essential promoter activity could be identified solely in the presence of this cap box element.

Of interest is the observation that altered DNA–protein binding specificities can be achieved by supplementing the growth media with different sera. Observations from different systems have shown that hormone binding can induce a conformational change in the oestrogen receptor, which leads to an increased affinity for DNA (McGrogan *et al.*, 1985; Skafar & Notides, 1985), while HBV X protein can alter the DNA-binding specificity of CREB and ATF-2 by protein–protein interactions (Maguire *et al.*, 1991). The precise effect of any given factor is determined by a variety of elements, such as cell type, concentration of factor and the duration of the stimulus, with the result that certain growth factors may fulfil quite different functions under different circumstances. Changing serum conditions might cause modifications in enzymes that act upon such elements, which in turn may influence the regulatory machinery. The above demonstration of serum-specific changes might mean that region A mediates transcriptional activation under certain conditions through serum stimulation. It is well known that serum induction of cell division requires *ras* protein and possibly *ras* expression, based on the fact that the levels of transcription of several other proto-oncogenes are increased soon after serum addition (Gauthier-Rouviere *et al.*, 1990; Mulcahy *et al.*, 1985). The *ras* protein may, therefore, represent a common element in the molecular cascade of events initiated by numerous growth factors referred to as serum components.

Since the *ras* genes are a ubiquitous gene family and likely to play a fundamental role in normal cellular function based on their high degree of conservation throughout eukaryotic evolution, it is not unexpected that their regulatory mechanisms may have been conserved to some degree as well.

We are grateful to Professor Igor Dawid for providing the *Drosophila* S2 cells, and to Professor Pierre Chambon and Professor Dawid Botes for synthesising the oligomers. We thank Dr Zeev Lev and co-workers for the use of the promoter deletion constructs and Dr Yamaguchi for his useful comments. Thanks are also due to Dr Zeev Lev and Professor Barry Fabian for the critical reading of the manuscript. This work was supported by the South African Medical Research council, the Foundation for Research and Development and Griffin Cancer Trust.

### References

ALITALO, K. (1984). Amplification of cellular oncogenes in cancer cells. *Med. Biol.*, **62**, 304–317.

ALMOGUERA, C., SHIBATA, D., FORRESTER, K., MARTIN, J., ARNHEIM, N. & PERUCHO, M. (1988). Most human carcinomas of the exocrine pancreas contain mutant c-K-ras genes. *Cell*, **53**, 549–554.

ARKHIPOVA, I.R. & ILYIN, Y.V. (1991). Properties of promoter regions of Mdgl *Drosophila* retrotransposon indicate that it belongs to a specific class of promoters. *EMBO J.*, **10**, 1169–1177.

BARBACID, M. (1987). Ras genes. *Annu. Rev. Biochem.*, **56**, 779–827.

BARBACID, M. (1990). Ras oncogenes: their role in neoplasia. *Eur. J. Clin. Invest.*, **20**, 225–235.

BAR-SAGI, D. & FERAMISCO, J.R. (1985). Microinjection of the ras oncogene protein into PC12 cells induces morphological differentiation. *Cell*, **42**, 841–848.

BIRCHMEIER, C., BROEK, D. & WIGLER, M. (1985). RAS proteins can induce miosis in *Xenopus* oocytes. *Cell*, **43**, 615–621.

BLIN, N., MULLER-BRECHLIN, R., CARSTENS, C., MEESE, E. & ZANG, K.D. (1987). Enhanced expression of 4 cellular oncogenes in a human glioblastoma cell-line. *Cancer Genet. Cytogenet.*, **25**, 285–292.

BOS, J.L. (1988). The ras gene family and human carcinogenesis. *Mutat. Res.*, **195**, 255–271.

BOS, J.L. (1989). Ras oncogenes in human cancer – a review. *Cancer Res.*, **49**, 4682–4689.

BOS, J.L., VERLAAN-DE VRIES, M., MARSHALL, C.J., VEENEMAN, G.H., VAN BOOM, J.H. & VAN DER EB, A.J. (1986). A human gastric carcinoma contains a single mutated and an amplified normal allele of the Ki-ras oncogene. *Nucleic Acids Res.*, **14**, 1209–1217.

BOS, J.L., FEARON, E.R., HAMILTON, S.R., VERLAAN-DE VRIES, M., VAN BOOM, J.H., VAN DER EB, A.J. & VOGELSTEIN, B. (1987). Prevalence of ras gene-mutations in human colorectal cancers. *Nature*, **327**, 293–297.

BROCK, H.W. (1987). Sequence and genomic structure of ras homologs Dmras85D and Dmras64B of *Drosophila melanogaster*. *Gene*, **51**, 129–137.

BUCHER, P. (1990). Weight matrix descriptions of 4 eukaryotic RNA polymerase II promoter elements derived from 502 unrelated promoter sequences. *J. Mol. Biol.*, **212**, 563–578.

BURBELO, P.D., MARTIN, G.R. & YAMADA, Y. (1988). 1(IV) and 2(IV) collagen genes are regulated by a bidirectional promoter and a shared enhancer. *Proc. Natl Acad. Sci. USA*, **85**, 9679–9682.

CHANG, E.H., FURTH, M.E., SCOLNICK, E.M. & LOWY, D.R. (1982). Tumorigenic transformation of mammalian cells induced by a normal human gene homologous to the oncogene of Harvey Murine Sarcoma virus. *Nature*, **297**, 479–483.

CHARDIN, P. (1988). The ras superfamily proteins. *Biochimie*, **70**, 865–868.

CHARDIN, P. (1991). Small GTP-binding proteins of the ras family – a conserved functional mechanism. *Cancer Cells*, **3**, 117–126.

COHEN, N., SALZBERG, A. & LEV, Z. (1988). A bidirectional promoter is regulating the *Drosophila* ras2 gene. *Oncogene*, **3**, 137–142.

DAVIDSON, I., FROMENTAL, C., AUGEREAU, P., WILDEMAN, A., ZENKE, M. & CHAMBON, P. (1986). Cell-type specific protein binding to the enhancer of simian virus 40 in nuclear extracts. *Nature*, **323**, 544–548.

DE-BIASI, F., DELSAL, G. & HAND, P.H. (1989). Evidence of enhancement of the ras oncogene protein product p21 in a spectrum of human tumors. *Int. J. Cancer*, **43**, 431–435.

DHAR, R., NIGTU, A., KOLLER, R., DEFEO-JONES, D. & SCOLNICK, E.M. (1984). Nucleotide sequence of two ras related-genes isolated from the yeast *Saccharomyces cerevisiae*. *Nucleic Acids Res.*, **12**, 3611–3618.

DIGNAM, J.D., LEBOVITZ, R.M. & ROEDER, R.G. (1983). Accurate transcription initiation by RNA polymerase II in a soluble extract from isolated mammalian nuclei. *Nucleic Acids Res.*, **11**, 1475–1489.

DOWNWARD, J. (1990). The ras superfamily of small GTP-binding proteins. *Trends Biol. Sci.*, **15**, 469–472.

EGAN, S.E., GIDDINGS, B.W., BROOKS, M.W., BUDAY, L., SIZELAND, A.M. & WEINBERG, R.A. (1993). *Nature*, **363**, 45–51.

FARNHAM, P.J., ABRAMS, J.M. & SCHIMKE, R.T. (1985). Opposite-strand RNAs from the 5′ flanking region of the mouse Dihydrofolate Reductase gene. *Proc. Natl Acad. Sci. USA*, **82**, 3978–3982.

FASANO, O., ALDRICH, T., TAMANOI, F., TAPAROWSKY, E., FURTH, M. & WIGLER, M. (1986). Analysis of the transforming potential of the human H-ras gene by random mutagenesis. *Proc. Natl Acad. Sci. USA*, **81**, 4008–4012.

FERAMISCO, J.R., GROSS, M., KAMATA, T., ROSENBERG, M. & SWEET, R.W. (1984). Microinjection of the oncogene form of the human H-ras (T24) protein results in rapid proliferation of quiscent cells. *Cell*, **38**, 109–117.

FORRESTER, K., ALMOGUERA, C., HAN, K., GRIZZLE, W.E. & PERUCHO, M. (1987). Detection of high-incidence of K-ras oncogenes during human colon tumorigenesis. *Nature*, **327**, 298–303.

GALLICK, G.E., KURZROCK, R., KLOETZER, W.S., ARLINGHAUS, R.B. & GUTTERMAN, J.U. (1985). Expression of $p21^{ras}$ in fresh primary and metastatic human colorectal tumors. *Proc. Natl Acad. Sci. USA*, **82**, 1795–1799.

GAUTHIER-ROUVIERE, C., FERNANDEZ, A. & LAMB, N.J.C. (1990). Ras-induced c-fos expression and proliferation in living rat fibroblasts involves c-kinase activation and the serum response element pathway. *EMBO J.*, **9**, 171–180.

GORMAN, C.M., MERLINO, G.T., WILLINGHAM, M.C., PASTAN, I. & HOWARD, B.H. (1982). Recombinant genomes which express chloramphenicol acetyltransferase in mammalian cells. *Mol. Cell. Biol.*, **2**, 1044–1051.

GROSSCHEDL, R. & BIRNSTIEL, M.L. (1980). Identification of regulatory sequences in the prelude sequence of an H2A histone gene by the study of specific deletion mutants in-vivo. *Proc. Natl Acad. Sci. USA*, **77**, 1432–1436.

HAGAG, N., HALEGONA, S. & VIOLA, M. (1986). Inhibition of growth factor-induced differentiation of Pc12 cells by microinjection of antibody to Ras p21. *Nature*, **319**, 680–682.

HALL, A. (1990). The cellular functions of small GTP binding proteins. *Science*, **249**, 635–640.

HALL, A. & BROWN, R. (1985). Human N-ras: cDNA cloning and gene structure. *Nucleic Acids Res.*, **13**, 5255–5268.

HAND, P.H., THOR, A., WUNDERLICH, D., MAURANO, R., CARUSO, A. & SCHLOM, J. (1984). Monoclonal antibodies of pre-defined specificity detect activated ras gene expression in human mammary and colon carcinomas. *Proc. Natl Acad. Sci. USA*, **81**, 5227–5231.

HARIHARAN, I.K., CARTHEW, R.W. & RUBIN, G.M. (1991). The *Drosophila* roughened mutation: activation of a rap homolog disrupts eye development and interferes with cell determination. *Cell*, **67**, 717–722.

HOFFMAN, E.K., TRUSKO, S.P., FREEMAN, N. & GEORGE, D.L. (1987). Structural and functional characterization of the promoter region of the mouse c-K-ras gene. *Mol. Cell. Biol.*, **7**, 2592–2596.

HUNTER, T. (1991). Cooperation between oncogenes. *Cell*, **64**, 249–270.

ISHII, S., MERLINO, G.T. & PASTAN, I. (1985*a*). Promoter region of the human Harvey ras proto-oncogene: similarity to the EGF receptor proto-oncogene promoter. *Science*, **230**, 1378–1381.

ISHII, S., XU, Y.-H., STRATTON, R.H., ROE, B.A., MERLINO, G.T. & PASTAN, I. (1985*b*). Characterization and sequence of the promoter region of the human epidermal growth factor receptor gene. *Proc. Natl Acad. Sci. USA*, **82**, 4920–4924.

JONES, N. (1990). Transcriptional regulation by dimerization – 2 sides to an incestuous relationship. *Cell*, **61**, 9–11.

KITAYAMA, H., SUGIMOTO, Y., MATSUZAKI, T., IKAWA, Y. & NODA, M. (1989). A ras-related gene with transformation suppressor activity. *Cell*, **56**, 77–84.

LEV, Z., KIMCHIE, Z., HESSLE, R. & SEGEV, O. (1985). Expression of ras cellular oncogenes during development of *Drosophila melanogaster*. *Mol. Cell. Biol.*, **5**, 1540–1542.

LEVANON, D., LIEMAN-HURWITS, J., DAFNI, N., WIGDERSON, M., SHERMAN, L., BERNSTEIN, Y., LAVER-RUDICH, Z., DANCIGER, E., STEIN, O. & GRONER, Y. (1985). Architecture and anatomy of the chromosomal locus in human chromosome 21 encoding the Cu/Zn superoxide dismutase. *EMBO J.*, **4**, 77–84.

LEWIN, B. (1991). Oncogenic conversion by regulatory changes in transcription factors. *Cell*, **64**, 303–312.

LIVEN, E., GLAZER, L., SEGAL, D., SCHLESSINGER, J. & SHILO, B.-Z. (1985). The *Drosophila* EGF receptor gene homology: conservation of both hormone binding and kinase domains. *Cell*, **40**, 599–607.

LOWNDES, N.F., PAUL, J., WU, J. & ALLAN, M. (1989). c-Ha-ras gene bidirectional promoter expressed in-vitro location and regulation. *Mol. Cell. Biol.*, **9**, 3758–3770.

MCCORMICK, F. (1989). Ras GTPase activating protein – signal transmitter and signal terminator. *Cell*, **56**, 5–8.

MCGRATH, J.P., CAPON, D.J., GOEDDEL, D.V. & LEVINSON, A.D. (1984). Comparative biochemical properties of normal and activated human ras p21 protein. *Nature*, **310**, 644–649.

MCGROGAN, M., SIMONSEN, C.C., SMOUSE, O.T., FARNHAM, P.J. & SCHIMKE, R.T. (1985). Heterogeneity at the estrogen receptor's affinity for DNA by estradiol. *J. Biol. Chem.*, **260**, 2307–2314.

MCMURRAY, C.T., WILSON, W.D. & DOUGLASS, J.O. (1991). Hairpin formation within the enhancer region of the human enkephalin gene. *Proc. Natl Acad. Sci. USA*, **88**, 666–670.

MAGUIRE, H.F., HOEFFLER, J.P. & SIDDIQUI, A. (1991). HBV X-protein alters the DNA-binding specificity of Creb and ATF-2 by protein–protein interactions. *Science*, **252**, 842–844.

MANIATIS, T., FRISCH, E. & SAMBROOK, J. (1989). Purification of plasmid DNA. *Molecular Cloning: A Laboratory Manual*, pp. 42–46. Cold Spring Harbor Laboratory Press: Cold Spring Harbor, NY.

MASTERS, J.N. & ATTARDI, G. (1985). Human dihydrofolate reductase gene transcripts present in polysomal RNA map with their 5′ ends several hundred nucleotides upstream of the main mRNA start site. *Mol. Cell. Biol.*, **5**, 493–500.

MAXAM, A.M. & GILBERT, W. (1980). Sequencing end-labeled DNA with base-specific chemical cleavages. *Methods Enzymol.*, **65**, 499–560.

MOZER, B., MARLOR, R., PARKHURST, S. & CORCES, V. (1985). Characterization and developmental expression of the *Drosophila* ras oncogene. *Mol. Cell. Biol.*, **5**, 885–889.

MULCAHY, L.S., SMITH, M.R. & STACEY, D.W. (1985). Requirement for ras proto-oncogene function during serum-stimulated growth of NIH 3T3 cells. *Nature*, **313**, 241–243.

NAKAJIMA, N., HORIKOSHIM, M. & ROEDER, R.G. (1988). Factors involved in specific transcription by mammalian RNA polymerase II – purification, genetic specificity, and TATA-box promoter interactions of TFIID. *Mol. Cell. Biol.*, **8**, 4028–4040.

NEUMAN-SILBERBERG, F.S., SCHEJTER, E., HOFFMANN, F.M. & SHILO, B.-Z. (1984). The *Drosophila* ras oncogenes: structure and nucleotide sequence. *Cell*, **37**, 1027–1033.

OSBORNE, T.F., GOLDSTEIN, J.L. & BROWN, M.S. (1985). 5′ end of HMG CoA reductase gene contains sequences responsible for cholesterol-mediated inhibition of transcription. *Cell*, **42**, 203–212.

PAPAGEORGE, A.G., DE FEO-JONES, D., ROBINSON, P., TEMEMLES, G. & SCOLNICK, E.M. (1984). *Saccharomyces cerevisiae* synthesises proteins related to the p21 gene product of ras genes found in mammals. *Mol. Cell. Biol.*, **4**, 23–29.

QNINONES, S., SAUS, J., OTANI, Y., HARRIS, E.D. & KURKINEN, M. (1989). Transcriptional regulation of human stromelysin. *J. Biol. Chem.*, **264**, 8339–8344.

REDDY, E.B., REYNOLDS, R.K., SANTOS, E. & BARBACID, M. (1982). A point mutation is responsible for the acquisition of tranforming properties by the T24 human bladder carcinoma oncogene. *Nature*, **300**, 149–152.

REYMOND, C.D., GOMER, R.H., MEHDY, M.C. & FIRTEL, R.A. (1984). Developmental regulation of a Dictyostelium gene encoding a protein homologous to mammalian RAS protein. *Cell*, **39**, 141–148.

RUBIN, G.M. (1991). Signal transduction and the fate of the R7 photoreceptor in *Drosophila*. *Trends Genet.*, **7**, 372–376.

RYAN, W.A., FRANZA, B.R. & GILMAN, M.Z. (1989). 2 distinct cellular phosphoproteins bind to the c-fos serum response element. *EMBO J.*, **8**, 1785–1792.

SALZBERG, A., COHEN, N., HALACHMI, N., KIMCHIE, Z. & LEV, Z. (1993). The Drosophila-Ras2 and Rop gene pair – a dual homology with a yeast Ras-like gene and a suppressor of its loss-of-function phenotype. *Development*, **117**, 1309–1319.

SANTORO, C., MERMOD, N., ANDREWS, P.C. & TJIAN, R. (1988). Cloning of cDNAs encoding human CCAAT-box binding proteins: a family of transcription and replication factors. *Nature*, **334**, 218–224.

SCHEJTER, E.D. & SHILO, B.-Z. (1985). Characterization of functional domains of p21 ras by use of chimeric genes. *EMBO J.*, **4**, 407–412.

SCHNEIDER, I. (1972). Cell lines derived from late embryonic stages of *Drosophila melanogaster*. *J. Embryol. Exp. Morphol.*, **27**, 353–365.

SEGAL, D. & SHILO, B.-Z. (1986). Tissue localization of *Drosophila melanogaster* ras transcription during development. *Mol. Cell. Biol.*, **6**, 2241–2248.

SHILO, B.-Z. (1987). Proto-oncogenes in *Drosophila melanogaster*. *Trends Genet.*, **3**, 69–72.

SKAFAR, D.F. & NOTIDES, A.C. (1985). Modulation of the estrogen receptor's affinity for DNA by estradiol. *J. Biol. Chem.*, **260**, 12208–12213.

SLEIGH, M.J. (1986). A nonchromatographic assay for expression of the chloramphenicol acetyltransferase gene in eukaryotic cells. *Anal. Biochem.*, **156**, 251–256.

SPANDIDOS, D.A. (1987). The consequences of quantitative and qualitative changes of Harvey-ras gene expression in the process of carcinogenesis. *Anticancer Res.*, **7**, 991–996.

SPANDIDOS, D.A. & RIGGIO, M. (1986). Promoter and enhancer-like activity at the 5′-end of normal and T24 Ha-Ras1 genes. *FEBS Lett.*, **203**, 169–174.

STRUHL, K. (1987). Promoters, activator proteins, and the mechanism of trnascriptional initiation in yeast. *Cell*, **49**, 295–297.

SWAROOP, A., SUN, J.-W., PACO-LARSON, M.L. & GAREN, A. (1986). Molecular-organization and expression of the genetic-locus glued in *Drosophila melanogaster*. *Mol. Cell. Biol.*, **6**, 833–841.

TABIN, C.J., BRADLEY, S.M., BARGMANN, C.I., WEINBERG, R.A., PAPAGEORGE, A.G., SCOLNICK, E.M., DHAR, R., LOWY, D.R. & CHANG, E.H. (1982). Mechanism of activation of a human oncogene. *Nature*, **300**, 143–149.

TAMURA, T. & KATSUHIKO, M. (1991). Role of GC-rich motif in transcription regulation of the adenovirus type 2 IVa2 promoter which lacks typical TATA-box element. *FEBS*, **282**, 87–90.

TAKAHIRO, N., YOSHIO, U. & SHUNSUKE, I. (1990). Transcriptional control of the human Harvey ras proto-oncogene: role of multiple elements in the promoter region. *Gene*, **94**, 249–253.

TANAKA, T., SLAMON, D.J., BATTIFORA, H. & CLINE, M.J. (1986). Expression of p21 RAS oncoproteins in human cancers. *Cancer Res.*, **46**, 1465–1470.

TAPAROWSKY, E., SUARD, Y., FASANO, O., SHIMIZU, K., GOLDFARB, M. & WIGLER, M. (1982). Activation of the T24 bladder carcinoma transforming gene is linked to a single amino acid change. *Nature*, **300**, 762–765.

THEILLET, C., BRUNET, M., CALLAHAN, R., ESCOT, C., GEST, J., HUTZELL, P., LIDEREAU, R. & SCHLOM, J. (1986). Loss of a c-Ha-ras1 allele and aggressive human primary breast carcinomas. *Cancer Res.*, **46**, 4776–4781.

WADSWORTH, S.C., VINCENT, W.S. & BILODEAN-WENTWORTH, D. (1985). A *Drosophila* genomic sequence with homology to human epidermal growth factor receptor. *Nature*, **314**, 178–180.

WILLIAMS, T.J. & FRIED, M. (1986). The MES-1 murine enhancer element is closely associated with the heterogeneous 5′ ends of 2 divergent transcription units. *Mol. Cell. Biol.*, **6**, 4558–4569.

XIAO, J.H., DAVIDSON, I., ROSALES, R., FERRANDON, D., VIGNERON, M., MACCHI, M. RUFFENACH, F. & CHAMBON, P. (1987). One cell-specific and three ubiquitous nuclear proteins bind in vitro to overlapping motifs in the domain B1 of the SV40 enhancer. *EMBO J.*, **6**, 3005–3013.

YAMAGUCHI, M., HIROSE, F., NISHIDA, Y. & MATSUKAGE, A. (1991). Repression of the *Drosophila* proliferating – cell nuclear antigen gene promoter by zerknullt protein. *Mol. Cell. Biol.*, **11**, 4909–4917.

YU, C.Y., MOTAMED, K., CHEN, J., BAILEY, A.D. & SHEN, C.-K.J. (1991). The CACC-box upstream of human embryonic epsilon globin gene binds Sp1 and is a functional promoter element in-vitro and in-vivo. *J. Biol. Chem.*, **266**, 8907–8915.